# Vamifeport: Monography of the First Oral Ferroportin Inhibitor

**DOI:** 10.3390/jcm13185524

**Published:** 2024-09-18

**Authors:** Federica Pilo, Emanuele Angelucci

**Affiliations:** 1 Hematology and Transplant Center, Azienda Ospedaliera Brotzu, 09121 Cagliari, Italy; 2 Hematology and Cellular Therapy Center, IRCCS Ospedale Policlinico San Martino, 16132 Genoa, Italy; emanuele.angelucci@hsanmartino.it

**Keywords:** ferroportin, hepcidin, sickle cell disease, thalassemia

## Abstract

Over the last few years, several mechanisms that are involved in congenital diseases characterized by ineffective erythropoiesis have been described. Therefore, multiple new target drugs are being developed in preclinical models against the main regulators of normal erythropoiesis. Above all, the key mechanism that regulates systemic iron homeostasis, represented by the hepcidin–ferroportin axis, is considered to be the target for new therapies. The main hypothesis is that iron restriction, through blocking ferroportin (the unique iron transporter in mammals) in such diseases, ameliorates erythropoiesis. The action of vamifeport is different from the currently approved drugs in this setting since it acts straight on the ferroportin–hepcidin axis. The data presented in the sickle cell disease (SCD) Townes mouse model showed a preclinical proof-of-concept for the efficacy of oral ferroportin inhibitor. Vamifeport reduced hemoglobin concentration in red blood cells (RBCs) and diminished intravascular hemolysis and inflammation, improving hemodynamics and preventing vascular occlusive crises. On this basis, clinical trials were commenced in patients with SCD, non-transfusion-dependent (NTD) thalassemia and transfusion-dependent (TD) thalassemia. Preliminary data in NTD thalassemic patients also confirm the safety and efficacy in decreasing iron level. In conclusion, vamifeport represents a new option in the panorama of drugs targeting the hepcidin–ferroportin axis, but its efficacy is still under investigation as a single agent.

## 1. Background

Vamifeport is an oral ferroportin inhibitor under investigation in congenital or acquired anemias where iron homeostasis could interfere with a normal erythropoiesis, such as sickle cell disease and thalassemia. Supporting the hypothesis that, by blocking ferroportin transporter, secondary iron restriction could ameliorate erythropoiesis, preclinical studies have been started.

In the SCD mice model, iron deficiency, produced by either a low-iron diet (LID) or genetic dysfunction of the hypoxia-inducible factor-2α (HIF-2α), enhanced anemia and hemolytic markers [1].

The data presented in the Townes mouse SCD model showed important preclinical proof for the efficacy of oral ferroportin inhibitor vamifeport [2].

The action of vamifeport is different from the currently approved drugs in this setting, since it acts straight on the ferroportin–hepcidin axis.

### 1.1. Ferroportin–Hepcidin Axis

Cellular iron homeostasis is controlled by multiple steps. One of the main described systems involves iron regulatory proteins (IRPs) and the iron-responsive element (IRE). The IRP/IRE system regulates the expression of several mRNAs encoding proteins for iron acquisition, storage, utilization and export (ferroportin) [3]. Based on iron availability, this system modulates the expression of genes involved in iron homeostasis.

Instead, systemic iron metabolism is mainly regulated by the hepcidin–ferroportin axis; this cooperation is responsible for the iron cycle (uptake, utilization, storage and recycling) [4].

Ferroportin, recognized as the only iron exporter in mammals, is a membrane protein present in all cells. Ferroportin gene transcription and translation are regulated by a number of complex signals, principally directed post-translationally by hepcidin [5]. Hepcidin, by binding and blocking ferroportin via degradation or occlusion, achieves an inhibitory effect on iron release within the blood from storage cells [6].

Hepcidin production responds to different signals, e.g., bone marrow iron requirements, hypoxia, transferrin saturation, iron stores, and inflammation (through different signaling pathways explained elsewhere) [7].

Increased hepcidin expression limits iron absorption at the enterocyte level, through ferroportin blocking, while its drop leads to greater iron absorption and macrophage iron release [6,8].

Recently, another actor of iron homeostasis has been individuated—the erythroferrone (ERFE). This TNFα-like protein released by mature erythroblasts in conditions of increased erythropoiesis is considered the main candidate of erythropoiesis-induced hepcidin suppression [9].

Under physiological conditions, during stress erythropoiesis, anemia causes increased erythropoietin (EPO) secretion by the kidneys. EPO stimulates ERFE secretion, by erythroblast cells, thus inhibiting hepcidin transcription. When the hepcidin level is reduced, an increase in the uptake of iron is physiologically described, because of the upregulated ferroportin’s degradation, as well as a release of recycled iron from macrophages, resulting in adequate iron accessibility for erythropoiesis and a suitable compensatory erythropoiesis [10].

Instead, in congenital or acquired disease characterized by dyserythropoiesis, iron overload from hepcidin, rather than inhibited, appears to exacerbate ineffective erythropoiesis.

In fact, in these conditions, erythroblasts do not efficaciously differentiate into mature erythrocytes; as a result, excessive iron release in serum cannot be utilized by compensatory effective erythropoiesis, increasing plasma transferrin saturation [11].

### 1.2. Hepcidin–Ferroportin Axis Behavior in Dyserythropoietic Diseases

In anemias with ineffective erythropoiesis (like thalassemia and SCD), most erythroblasts do not effectively differentiate into mature erythrocytes, leading to anemia and boosted EPO production by the kidneys. Nevertheless, differently from physiological conditions, the described erythroblastic hyperplasia response does not correspond to a recovery from anemia [11].

In other words, iron is mobilized by increased ERFE production acting by controlling the hepcidin–ferroportin axis, but while erythrocyte production cannot increase, the over-available serum iron is not correctly utilized. The result is that when transferrin uptake capability is exceeded, non-transferrin-bound iron (NTBI) and labile plasma iron (LPI) appear to develop cellular iron toxicity [4].

For this reason, a modulation of iron homeostasis has been proposed as a target for therapies that improve ineffective erythropoiesis, thereby ameliorating anemia.

The supposition that iron restriction could improve the condition of anemia in dyserythropoietic disease is a key concept in the development of molecules acting as hepcidin mimetics or ferroportin inhibitors such as vamifeport.

Iron restriction reduces erythropoiesis, acting through the pathway of transferrin receptor 2 (TfR2) that sets the expression of EPO receptors. Collocated on the cell surface of the immature erythroid precursors, the TfR2-altered pathway leads to the resistance of the erythron to EPO. The consequence is to support cell differentiation instead of proliferation, resulting in a positive contribution to downgrading the level of ineffective erythropoiesis [12].

Assumed its crucial role in iron homeostasis, the hepcidin–ferroportin axis symbolizes a new appealing therapeutic target for β-thalassemia but also for SCD. In particular, the inhibition of the ferroportin function leading to iron restriction for erythropoiesis, represents a innovative opportunity to targeting the hepcidin–ferroportin regulation.

## 2. Vamifeport

Vamifeport (VIT-2763) is an oral ferroportin inhibitor specifically designed to target the hepcidin–ferroportin axis and enhance ineffective erythropoiesis [12]. It is also known as 2-[2-[2-(1h-benzimidazol-2-yl)ethylamino]ethyl]-n-[(3-fluoropyridin-2-yl)methyl]-1,3-oxazole-4-carboxamide (molecular formula: C_21_H_21_FN_6_O_2_; molecular weight: 408.4 g/mol).

**Figure 1:**
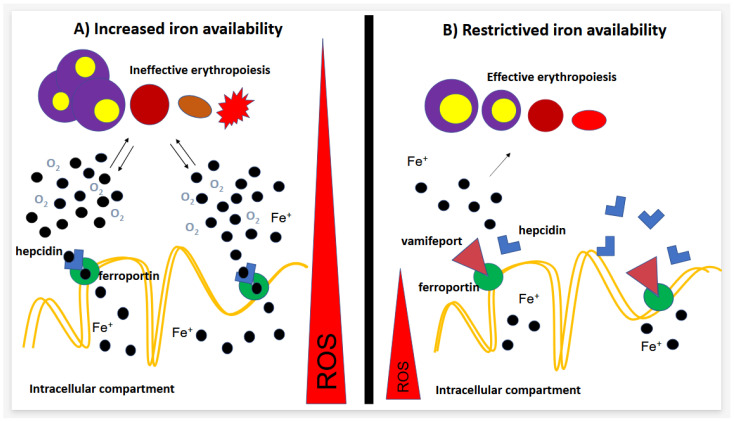
Schematic action of Vamifeport as modulator of iron availability for erythropoiesis.

Preclinical data demonstrated that vamifeport, as an inhibitor of ferroportin, acts in a similar way to hepcidin or, better, in competition with hepcidin in binding to ferroportin, dislocating hepcidin bound to recombinant ferroportin, and reducing cellular iron efflux with a potency similar to that of hepcidin (Figure 1) [13,14,15].

Vamifeport has been tested mostly in SCD. Mangoakar and colleagues have described how hepcidin is increased in sickle cell patients with iron overload when compared with sickle cell control patients [16].

Sickle cell disease is a genetic disease starting from a point mutation in the HBB gene that leads to the polymerization of hemoglobin S (HbS) during deoxygenation. HbS forms long chains of polymers that alter red blood cells (RBCs) into a sickle shape; this is the reason why the transit of these modify RBCs in smaller blood vessels, leading to hemolysis. Increased RBC lysis and release of free HbS generate nitric oxide (NO) and stimulate vasoconstriction, which further promotes the activation and mobilization of white blood cells (WBCs), increasing their adhesiveness to activated endothelium. These pathological signs in SCD finally result in painful vaso-occlusive crises, organ damage, and, rarely, premature death.

SCD is characterized by a relationship between inflammation, hemolysis and erythropoiesis that modulates the expression of hepcidin. In particular, hepcidin levels are inappropriately low compared to the iron levels presented, suggesting the existence of an additional and/or complementary inhibitory mechanism to ERFE. In sickle cell anemia, analyzing the relationship between inflammation and iron metabolism, it can be stated that inflammation (meaning increased hepcidin) protects patients with SCD by blocking iron within the reticuloendothelial system. Inflammation is less present in the other dyserytropoietic disease; therefore, the competitive regulation of hepcidin and its complete or partial consequences on iron balance in SCD remain difficult to interpret at the moment and need further investigation [17,18].

Unlike thalassemia intermedia, in non-transfused sickle cell patients, there is no evidence of a spontaneous accumulation of iron in target organs but they may even develop a condition of iron deficiency; overall, patients with SCD undergoing chronic transfusions appear to have a lower risk of developing iron-related complications compared to thalassemic patients and a lower distribution of iron in extrahepatic tissues [19].

### 2.1. Preclinical Studies in SCD and Thalassemia Model

The effect of vamifeport and hepcidin on ferroportin degradation was evaluated, for the first time in vitro, in a human breast cancer cell line (T47D); expressing ferroportin, T47D cells were incubated with iron sulfate characterized by the stable isotope 58Fe [13].

Both vamifeport and hepcidin acted by inhibiting the cellular iron efflux in a dose-dependent manner (hepcidin was less effective than vamifeport). Using J774 (Sukhi Bansal, King’s College London, London, UK), another cell line, the results were the same: vamifeport and hepcidin comparably triggered ferroportin internalization and degradation [13].

The authors also considered a mouse model in which a condition of a very low level of blood iron concentration was created. The oral administration of 60 mg/kg of vamifeport developed a rapid diminution in serum iron similar to that produced from hepcidin (40% and 58% lower, respectively) than the control. This result is also confirmed in anemic Wistar rats (nurtured with a low-iron diet to develop anemia). In this mouse, an oral iron dose was administered followed by vamifeport at 10 or 30 mg/kg. Vamifeport showed a significant serum iron suppression compared to the control [15].

To evaluate the impact of vamifeport on organ iron concentrations, a murine model of hereditary hemochromatosis (HFE C282Y) was used; vamifeport in this experiment also showed a reduction in serum iron to the levels of wild-type mice and avoided iron holding in the livers of the hemochromatosis mice model [15].

Very interesting results have been obtained with the SCD Townes mouse model, where vamifeport showed the following capabilities: reduced hemoglobin concentration in RBCs, diminished intravascular hemolysis, downgraded inflammation, improved hemodynamics, and also prevented vascular stasis. The hypothesis, postulated by the authors, is that vamifeport might reduce HbS concentration in RBCs and decrease corpuscular hemoglobin concentration mean and mean corpuscular volume, while at the same time increasing hypochromic and microcytic RBC fractions. To summarize, vamifeport in the SCD Townes mouse model decreased hemolysis, improved blood flow, and prevented vaso-occlusive events [2] but at the doses tested, vamifeport did not notably exacerbate anemia [2].

Having been demonstrated, in previous studies, a possible approach to lower RBC HbS concentration, inducing iron-restricted erythropoiesis, a HbSS mouse model explored the efficacy of vamifeport using these tools [20,21,22]. Vamifeport reduced RBC HbS concentrations (CHCM) of HbSS mice by ∼5%. Even if a small effect on HbS concentrations is considered, it might favor a substantial and clinically relevant delay in HbS polymerization upon deoxygenation, thereby preventing hemolysis and vaso-occlusion [20,21,22].

Vamifeport also showed a reduction in the spleen burden of HbSS mice (by 31% compared with HbSS control mice), indicating enhancement of the extramedullary erythropoiesis, further improving RBC membrane composition and mitochondria clearance. The proportion of mitochondria-containing mature RBCs in HbSS mice was lower compared with vehicle-treated control mice (18.8% ± 8.5%); basically, by ameliorating the clearance of mitochondria by vamifeport, the oxidative stress induced by hemolysis and eryptosis may be decreased [2].

It is well known that, in SCD, increased levels of circulating adhesion molecules lead to endothelial activation but vamifeport reduced systemic inflammation and lowered plasma soluble VCAM-1 (sVCAM-1) and soluble platelet selectin (sP-selectin) levels in HbSS mice.

Additionally, RBCs from HbSS mice treated with vamifeport presented superior deformability under hypoxic conditions, revealing a beneficial effect of vamifeport on hypoxia-induced severity of sickling, preventing vaso-occlusion in the Townes model of SCD [2].

Regarding thalassemia, the effects of vamifeport have been studied using the Hbbth3/+ murine model of NTDT (not transfusion-dependent thalassemia) [13].

As for SCD, as well as in the thalassemia mouse model, vamifeport dosed at 30 and 100 mg/kg showed the capacity to significantly decrease serum iron levels compared with control (by 77% and 84%, respectively) and a reduction in relative spleen weight. Furthermore, there were decreased organ iron levels, demonstrating enhancement of anemia and erythropoiesis.

Essentially, vamifeport, reducing the amount of early erythroid precursors in the bone marrow and spleen, and increasing the number of mature erythrocytes, showed a more successful and effective erythropoiesis. Anemia is also improved by the dose-dependent ability of vamifeport to reduce the level of reactive oxygen species (ROS), hence tissue oxygenation, and to extend the lifespan of RBCs, thus improving anemia [13].

Taking these preclinical results into account altogether, the challenging issue in human models will be to obtain the right equilibrium between iron-restricted, meaning “beneficial”, and iron-deficient, meaning “damaging”, to obtain an effective erythropoiesis.

### 2.2. Clinical Studies

The data presented in the Townes mouse SCD model showed a preclinical proof-of-concept for the oral ferroportin inhibitor, vamifeport.

These data are fundamental to the development of protocols in humans—not only in mouse models.

The safety, tolerability, pharmacokinetics and dynamics of vamifeport in humans were first tested in 72 healthy adult volunteers in a Phase I, randomized, double-blind, placebo-controlled, multiple-ascending doses of vamifeport study [12]. Participants received single oral doses of vamifeport (5, 15, 60, 120, or 240 mg) or placebo in the single-ascending dose phase and multiple oral doses of vamifeport (60 or 120 mg once daily or twice daily), or placebo for 7 days in the multiple-ascending dose phase.

Vamifeport, both at single oral doses (up to 240 mg) or multiple oral doses (up to 120 mg twice daily), compared with placebo, was very well tolerated. The reduction in serum iron levels was rapid and transitory, observed in single doses ≥60 mg and all multiple doses. Mean calculated transferrin saturation also temporarily decreased with multiple doses of vamifeport (maximally at about 4–8 h after the morning dose). Severe adverse events (AEs) or discontinuations due to AEs were not reported in the healthy population in the study.

On this basis, clinical trials have commenced in patients with SCD (clinicaltrials.gov NCT04817670), NTD thalassemia (clinicaltrials.gov NCT04364269), and TD thalassemia (clinicaltrials.gov NCT04938635) [23] (Table 1).

Results of the phase 2, multiple-dose, double-blind, randomized, placebo-controlled study (VITHAL) in not transfusion-dependent beta-thalassemia (NTDT) has been presented at the European Hematology Association (EHA) annual meeting 2023 [24].

Twenty-five patients were included; the primary objective was to evaluate the safety and tolerability of vamifeport (assumed once (QD) or twice (BID) daily) versus placebo in patients with NTDT over a 12-week treatment period and results revealed no differences between groups in adverse events rates (vamifeport QD = 66%, BID = 58%; placebo, 75%). Thus, all AEs were mild or moderate intensity. No deaths or serious AEs were reported (only 1 patient receiving vamifeport QD had an acute hemolytic event). A secondary objective was to estimate pharmacodynamic effects on iron parameters (serum iron, ferritin, hepcidin and TSAT).

On day 1, after the first vamifeport dose, serum iron concentrations decreased after 3 h in all vamifeport-treated patients and mean (SD) TSAT also diminished on day 1 (3 h post dose), dropping to baseline levels 3–4 h post-dose at each successive visit in the vamifeport groups. Hematological markers do not report significant changes (no improvement of anemia or change in MCV, MCH and hepcidin level).

Authors declared that the limitations of the study were the small sample, short period of observation and heterogeneous population. Furthermore, they postulated that the model for improving erythropoiesis by inducing iron restriction may not be easily achievable in patients with pre-existing iron overload (Table 2).

## 3. Pharmacokinetics

As previously reported, pharmacokinetics studies have also showed how vamifeport in rodents acted in terms of reducing iron release into the plasma. Considering a mouse model of acute hypoferremia (C57BL/6 mice), authors showed how the oral administration of 60 mg/kg of vamifeport produced a rapid decrease in serum iron at 1 and 3 h post-dose and this effect was comparable to the hepcidin action in the same physiological condition. It was observed that the minimum serum iron levels were achieved after 4 h, and restored within 8–24 h [2].

The absorption of vamifeport was tested in the Phase I, randomized, double-blind, placebo-controlled study in 72 healthy adult volunteers; vamifeport was observed at detectable levels 15–30 min post-dose. No evidence of drug accumulation after 7 days of continual dosing in the multiple-dose group was reported. The vamifeport half-life (on day 1) was 1.9–5.3 h in the single-ascending dose phase, and (on day 7) 2.1–3.8 h and 2.6–5.3 h in the multiple-ascending dose phase [12].

## 4. Safety

No serious or severe adverse events (AEs) or withdrawals due to AEs were reported in the Phase I, randomized, double-blind, placebo-controlled study that enrolled 72 healthy adult volunteers [12].

In the VITHAL trials no deaths or serious AEs have been reported (one patient receiving vamifeport QD had an acute hemolytic event); one TEAE (increased creatine kinase and transaminase levels, not drug-related) led to drug discontinuation in the vamifeport BID group [24].

## 5. Drug Interactions

The relationship between vamifeport and the oral iron chelator deferasirox is a key point under further investigation. In a mouse model of beta-thalassemia, Nyffenegger and colleagues demonstrated no negative impact of vamifeport on the efficacy of deferasirox and vice versa [2].

As previously described, vamifeport alone decreased serum iron but did not decrease the overload of liver iron as it did with deferasirox [14]. Instead, the efficacy of vamifeport, alone or in combination with deferasirox, in terms of increasing hemoglobin, RBC counts, and hematocrit, and decreasing reticulocyte numbers, has been described; thus, it has the capability to decrease levels of ROS production and RBC apoptosis and to improve mitochondrial functions [14].

## 6. Conclusions

Vamifeport is an oral ferroportin inhibitor which is, at this moment, under investigation in thalassemia and SCD. It has a mode of action different from the currently approved drugs in this setting because it acts on the ferroportin–hepcidin axis, the key actor of iron’s homeostasis regulation of erythropoiesis. Generally speaking, iron restriction through blocking ferroportin should ameliorate erythropoiesis. In the thalassemia mouse model, vamifeport decreased serum iron levels, thus improving hematological parameters, including hemoglobin level and RBC count.

Preclinical data in the SCD Townes mouse model also demonstrated that vamifeport reduced hemoglobin concentration in RBCs and diminished intravascular hemolysis and inflammation, improving hemodynamics and preventing vascular occlusive crises.

Preliminary data in NTDT patients confirm its safety and efficacy in terms of decreasing iron level but do not show significant improvements in hematological markers; data regarding SCD are ongoing but the primary end point is different from thalassemia trials, which is, namely, the amelioration of hemolytic markers (as demonstrated in the SCD mouse model).

In conclusion, vamifeport is a new option in the panorama of drugs targeting the hepcidin–ferroportin axis, but its efficacy is still under investigation as a single agent. It will be particularly interesting in the future to understand if it could be more useful in SCD rather than in thalassemia and which will be the best partner for vamifeport.

In our opinion, vamifeport should be a perfect partner for the new target drugs acting in later stages of erythropoiesis such as the novel inhibitors of the TGF-ß (tumor growth factor) pathway Luspatercept and KER-050, or for the activators of erythrocyte pyruvate kinase such as Mitapivat. We also consider vamifeport a challenge and explorable opportunity in other acquired dyserithropoietic diseases such as myelodysplastic syndrome.

## Figures and Tables

**Table 1:** Completed and ongoing studies.

Trial	Phase	Target Population	Objectives	Clinicaltrial.gov ID	Status
VITHAL	Phase 2a, double-blind, randomized, placebo-controlled, parallel group, multicenter study on safety, tolerability, pharmacokinetics, pharmacodynamics and preliminary efficacy of multiple doses of VIT-2763 in subjects with non-transfusion-dependent β-thalassemia	People aged 18 and older with NTDT 12 years to 65 years	Tolerability and safety.	NCT04364269	Completed
VIT-2763-THAL-203	Phase 2b multiple-dose, double-blind, randomized, placebo-controlled, parallel-group, multicenter trial	TDT	The main purpose of this study is to evaluate the efficacy of 3 multiple doses of VIT-2763 as measured by the reduction in red blood cell (RBC) transfusion burden from week 13 to week 24, to identify the most efficacious and safe dose.	NCT04938635	Withdrawn (strategic reasons)
VIT-2763-SCD-202	Phase 2a, double-blind, randomized, placebo-controlled, efficacy, and safety study of multiple doses of VIT-2763 in subjects with sickle cell disease	SCD	The purpose of this study is to investigate the effect of VIT-2763 on markers of hemolysis (breakdown in red blood cells) in sickle cell disease (SCD). The safety, tolerability and clinical beneficial effects of VIT-2763 for the treatment of SCD are also explored.	NCT04817670	Ongoing

**Table 2:** Summary of preclinical and clinical results for vamifeport.

	Preclinical Results	Clinical Results
Thalassemia	Reduced organ iron levels. Reduced the level of reactive oxygen species (ROS). Reduced the amount of early erythroid precursors in the bone marrow and spleen, and increased the number of mature erythrocytes. Ameliorated hematological parameters.	Safety and tolerability (revealed no differences between groups in adverse events rates (vamifeport, QD = 66%, BID = 58%; placebo, 75%)). All AEs were mild or moderate intensity. No deaths or serious AEs were reported. Decreased serum iron concentrations. Hematological markers do not report significant changes.
SCD	Reduced organ iron levels. Reduced HbS concentration in RBCs and decreased corpuscular hemoglobin concentration mean and mean corpuscular volume, delaying polymerization upon deoxygenation, therefore preventing not only hemolysis of circulating RBCs but also vaso-occlusion. Improved hemodynamics and also prevented vascular stasis. Reduced the spleen weight of HbSS mice, indicating enhancement of the extramedullary erythropoiesis. Improved RBC membrane composition and mitochondria clearance. Lowered plasma soluble VCAM-1 (sVCAM-1) and soluble platelet selectin (sP-selectin) levels in HbSS mice, resulting in reduced systemic inflammation.	Study ongoing

## References

[1] Parrow N.L., Violet P.-C., George N.A., Ali F., Bhanvadia S., Wong R., Tisdale J.F., Fitzhugh C., Levine M., Thein S.L. (2021). Dietary iron restriction improves markers of disease severity in murine sickle cell anemia. Blood.

[2] Nyffenegger N., Zennadi R., Kalleda N., Flace A., Ingoglia G., Buzzi R.M., Doucerain C., Buehler P.W., Schaer D.J., Dürrenberger F. (2022). The oral ferroportin inhibitor vamifeport improves hemodynamics in a mouse model of sickle cell disease. Blood.

[3] Muckenthaler M.U., Galy B., Hentze M.W. (2008). Systemic iron homeostasis and the iron-responsive element/iron-regulatory protein (IRE/IRP) regulatory network. Annu. Rev. Nutr..

[4] Pilo F., Cilloni D., Della Porta M.G., Forni G.L., Piperno A., Santini V., Angelucci E. (2022). Iron-mediated tissue damage in acquired ineffective erythropoiesis disease: It’s more a matter of burden or more of exposure to toxic iron form?. Leuk. Res..

[5] Drakesmith H., Nemeth E., Ganz T. (2015). Ironing out Ferroportin. Cell Metab..

[6] Aschemeyer S., Qiao B., Stefanova D., Valore E.V., Sek A.C., Ruwe T.A., Vieth K.R., Jung G., Casu C., Rivella S. (2018). Structure-function analysis of ferroportin defines the binding site and an alternative mechanism of action of hepcidin. Blood.

[7] Muckenthaler M.U., Rivella S., Hentze M.W., Galy B. (2017). A Red Carpet for Iron Metabolism. Cell.

[8] Nemeth E., Tuttle M.S., Powelson J., Vaughn M.B., Donovan A., Ward D.M., Ganz T., Kaplan J. (2004). Hepcidin regulates cellular iron efflux by binding to ferroportin and inducing its internalization. Science.

[9] Kautz L., Jung G., Valore E.V., Rivella S., Nemeth E., Ganz T. (2014). Identification of erythroferrone as an erythroid regulator of iron metabolism. Nat. Genet..

[10] Coffey R., Ganz T. (2018). Erythroferrone: An Erythroid Regulator of Hepcidin and Iron Metabolism. HemaSphere.

[11] Ganz T. (2019). Erythropoietic regulators of iron metabolism. Free Radic. Biol. Med..

[12] Richard F., van Lier J.J., Roubert B., Haboubi T., Göhring U.-M., Dürrenberger F. (2020). Oral ferroportin inhibitor VIT-2763: First-in-human, phase 1 study in healthy volunteers. Am. J. Hematol..

[13] Manolova V., Nyffenegger N., Flace A., Altermatt P., Varol A., Doucerain C., Sundstrom H., Dürrenberger F. (2019). Oral ferroportin inhibitor ameliorates ineffective erythropoiesis in a model of β-thalassemia. J. Clin. Investig..

[14] Nyffenegger N., Flace A., Doucerain C., Dürrenberger F., Manolova V. (2021). The Oral Ferroportin Inhibitor VIT-2763 Improves Erythropoiesis without Interfering with Iron Chelation Therapy in a Mouse Model of β-Thalassemia. Int. J. Mol. Sci..

[15] Nyffenegger N., Flace A., Canclini C., Duerrenberger F., Manolova V. (2017). Ferroportin inhibitors prevent iron loading in a mouse model of hereditary hemochromatosis. Am. J. Hematol..

[16] Mangaonkar A.A., Thawer F., Son J., Ajebo G., Xu H., Barrett N.J., Wells L.G., Bowman L., Clair B., Patel N. (2020). Regulation of iron homeostasis through the erythroferrone-hepcidin axis in sickle cell disease. Br. J. Haematol..

[17] Ginzburg Y.Z., Glassberg J. (2018). Inflammation, Hemolysis, and Erythropoiesis Lead to Competitive Regulation of Hepcidin and Possibly Systemic Iron Status in Sickle Cell Disease. EBioMedicine.

[18] Ezeh C., Ugochukwu C.C., Weinstein J., Okpala I. (2005). Hepcidin, haemoglobin and ferritin levels in sickle cell anaemia. Eur. J. Haematol..

[19] Wood J.C., Cohen A.R., Pressel S.L., Aygun B., Imran H., Luchtman-Jones L., Thompson A.A., Fuh B., Schultz W.H., Davis B.R. (2016). TWiTCH Investigators Organ iron accumulation in chronically transfused children with sickle cell anaemia: Baseline results from the TWiTCH trial. Br. J. Haematol..

[20] Lincoln T.L., Aroesty J., Morrison P. (1973). Iron-deficiency anemia and sickle-cell disease: A hypothesis. Lancet.

[21] Hofrichter J., Ross P.D., Eaton W.A. (1974). Kinetics and mechanism of deoxyhemoglobin S gelation: A new approach to understanding sickle cell disease. Proc. Natl. Acad. Sci. USA.

[22] Castro O., Poillon W.N., Finke H., Massac E. (1994). Improvement of sickle cell anemia by iron-limited erythropoiesis. Am. J. Hematol..

[23] Porter J., Taher A., Viprakasit V., Kattamis A., Coates T.D., Garbowski M., Dürrenberger F., Manolova V., Richard F., Cappellini M.D. (2021). Oral ferroportin inhibitor vamifeport for improving iron homeostasis and erythropoiesis in β-thalassemia: Current evidence and future clinical development. Expert Rev. Hematol..

[24] Taher A., Kourakli-Symeonidis A., Tantiworawit A., Wong P., Szecsödy P. (2022). S272: Safety and preliminary pharmacodynamic effects of the Ferroportin inhibitor Vamifeport (VIT-2763) in patients with non-transfusion-dependent Beta Thalassemia (NTDT): Results from a phase 2A study. HemaSphere.

